# Improved Overall Survival of Colorectal Cancer under Multidisciplinary Team: A Meta-Analysis

**DOI:** 10.1155/2021/5541613

**Published:** 2021-05-01

**Authors:** Dong Peng, Yu-Xi Cheng, Yong Cheng

**Affiliations:** Department of Gastrointestinal Surgery, The First Affiliated Hospital of Chongqing Medical University, Chongqing, China 400016

## Abstract

**Purpose:**

The purpose of the current meta-analysis was to evaluate whether multidisciplinary team improved overall survival of colorectal cancer.

**Methods:**

PubMed, EMBASE, and Cochrane Library database were searched from inception to October 25, 2020. The hazard ratio (HR) and 95% confidence (CI) of overall survival (OS) were calculated.

**Results:**

A total of 11 studies with 30814 patients were included in this meta-analysis. After pooling the HRs, the MDT group was associated with better OS compared with the non-MDT group (HR = 0.81, 95% CI 0.69-0.94, *p* = 0.005). In subgroup analysis of stage IV colorectal cancer, the MDT group was associated with better OS as well (HR = 0.73, 95% CI 0.59-0.90, *p* = 0.004). However, in terms of postoperative mortality, no significant difference was found between MDT and non-MDT groups (OR = 0.84, 95% CI 0.44-1.61, *p* = 0.60).

**Conclusion:**

MDT could improve OS of colorectal cancer patients.

## 1. Introduction

Colorectal cancer ranks the third largest cancer in the world and the second leading cause of cancer-related death, resulting in approximately 600,000 deaths each year [1, 2]. Metastases occur in 20% to 30% of patients at the time of diagnosis [3, 4]. Surgery is the main treatment for patients with colorectal cancer; in addition, other treatment including radiotherapy and chemotherapy can improve the prognosis as well [5–7].

The core of the multidisciplinary team (MDT) is a patient-centered focus model by regularly organizing experts in related disciplines to discuss and make the most suitable treatment plan for patients [8]. MDT has proven to be effective in the treatment of breast cancer, oral cancer, and prostate cancer [9–11]. In recent years, MDT has become an increasingly popular form of diagnosis and treatment in colorectal cancer [12, 13]. The MDT of colorectal cancer is composed of experts in multiple fields including surgery, oncology, radiology, pathology, and other related disciplines [14, 15].

Previous studies reported survive benefit of MDT on colorectal cancer [16, 17]; however, other studies held negative point [18, 19]. Therefore, the purpose of the current meta-analysis was to evaluate whether MDT improved OS of colorectal cancer.

## 2. Methods

The current meta-analysis followed the Preferred Reporting Items for Systematic Reviews and Meta-Analyses (PRISMA) statement [20].

### 2.1. Literature Search Strategy

PubMed, EMBASE, and Cochrane Library database were searched by two authors, respectively. The literature search was performed on October 25, 2020. The search strategy focused on two items including multidisciplinary team and colorectal cancer. For the multidisciplinary team, the search strategy was as follows: (multidisciplinary) OR (multidisciplinary) OR (MDT). For colorectal cancer, the search strategy was as follows: (colorectal cancer) OR (colon cancer) OR (rectal cancer) OR (colorectal neoplasm) OR (colon neoplasm) OR (rectal neoplasm) OR (colorectal tumor) OR (colon tumor) OR (rectal tumor). After that, we combined the two search items using “AND,” and the scope of search strategy was limited in title and abstract.

### 2.2. Inclusion and Exclusion Criteria

The screening of the studies was carried out by two authors independently. Initially, titles and abstracts were screened to exclude irrelevant studies, and after that, full texts were required for further screening. Case reports, nonhuman studies, conference abstracts, comments, and letters to editor were excluded. All included studies were checked for duplicate medical records from the same or overlapping cohort of patients. Finally, all the included studies were crosschecked by the two authors.

### 2.3. Data Extraction and Quality Assessment

The data were extracted by two authors. The extracted data included the first author, publishing year, country, study design, study date, sample size, sex, tumor location, and HRs with associated 95% CIs or *p* value for OS. HRs were extracted from multivariate analyses and/or univariate analyses or estimated from Kaplane-Meier survival curves [21, 22]. Any discrepancies between the two authors were resolved by a third author for consensus. The quality of the included studies was assessed based on the Newcastle-Ottawa Scale (NOS) [23], and a NOS score ≥ 7 was considered high-quality studies [24].

### 2.4. Statistical Analysis

Pooled HRs and 95%CIs were calculated for OS of colorectal cancer. For dichotomous variables, odds ratios (ORs) were calculated, respectively. 95% confidence intervals (CI) were calculated. The value of *I*^2^ and the result of the Chi-squared test were used to assess the statistical heterogeneity [25, 26]. It was considered high heterogeneity when *I*^2^ > 50%, the random effect model was used, and *p* < 0.1 was considered statistically significant. While the fixed effect model was used for the studies with *I*^2^ ≤ 50%, *p* < 0.05 was considered statistically significant. This meta-analysis was performed with RevMan 5.3 (The Cochrane Collaboration, London, United Kingdom).

## 3. Result

### 3.1. Search Results

**Figure 1:**
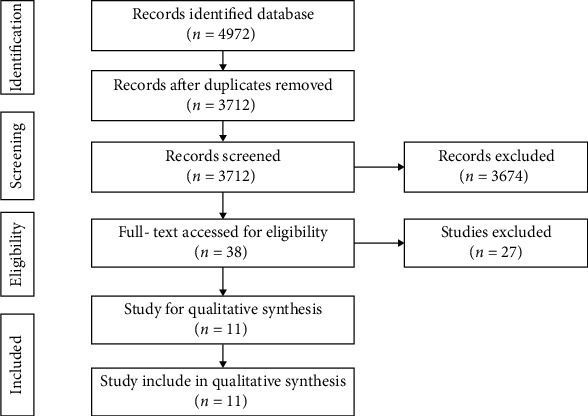
Flowchart of study selection.

A total of 4972 studies were screened in database, and eleven studies [16–19, 27–33] with 30814 patients were included for meta-analysis according to the inclusion and exclusion criteria, and the flowchart of study selection was shown in Figure 1.

### 3.2. Characteristics of the Included Studies

The 11 studies were published from 2011 to 2020, seven of which were from China, and the others were from The Netherlands, Australia, Denmark and The United Kingdom, respectively. There were 6 retrospective studies and 5 cohort studies. The sample size, sex, tumor stage and NOS score were shown in Table 1.

### 3.3. Impact of MDT on Overall Survival

**Figure 2:**
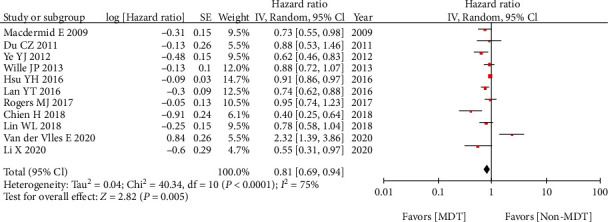
Impact of MDT on overall survival.

All the 11 studies reported MDT on OS. After pooling the HRs, a significant difference was found between the MDT group and non-MDT group (HR = 0.81, 95% CI 0.69-0.94, *p* = 0.005). These results suggested that the MDT group was associated with better OS (Figure 2).

### 3.4. Subgroup Analysis of MDT on Overall Survival of Stage IV Colorectal Cancer

**Figure 3:**
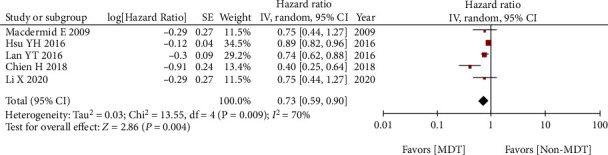
Subgroup analysis of MDT on overall survival of stage IV colorectal cancer.

Five studies reported the HRs of stage IV colorectal cancer, and in order to explore MDT on OS of the specific tumor stage, we performed subgroup analysis of stage IV colorectal cancer. The MDT group was associated with better OS compared with the non-MDT group in terms of stage IV colorectal cancer (HR = 0.73, 95% CI 0.59-0.90, *p* = 0.004) (Figure 3).

### 3.5. Impact of Other Factors on Overall Survival

There were other factors reported influencing OS, and we conducted subgroup analysis of the factors including sex, age, differentiation, tumor stage, and neoadjuvant chemotherapy. After pooling up the HRs, old age (HR = 1.38, 95% CI 1.09-1.74, *p* = 0.006), poor differentiation (HR = 1.35, 95% CI 1.06-1.72, *p* = 0.01), and higher tumor stage (HR = 1.77, 95% CI 1.19-2.65, *p* = 0.005) were related to poor OS. The results of subgroup analysis were shown in Table 2.

### 3.6. Postoperative Mortality between MDT and Non-MDT Groups

**Figure 4:**
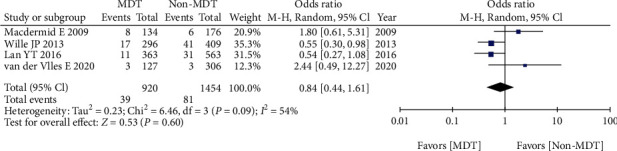
Postoperative mortality between MDT and non-MDT groups.

Four studies reported the postoperative mortality between MDT and non-MDT groups. After pooling up all the data, no significant difference was found between MDT and non-MDT groups (OR = 0.84, 95% CI 0.44-1.61, *p* = 0.60) (Figure 4).

## 4. Discussion

A total of 11 studies with 30814 patients were included in this meta-analysis. After pooling the HRs, the MDT group was associated with better OS compared with the non-MDT group. In subgroup analysis of stage IV colorectal cancer, the MDT group was associated with better OS as well. However, in terms of postoperative mortality, no significant difference was found between MDT and non-MDT groups.

Despite the reduction in mortality with changes in screening tests and lifestyle, the prognosis of patients with metastatic colorectal cancer was still poor [34, 35]. There were many prognostic factors including tumor stage, tumor location, and obesity which could affect the OS [36, 37]. In this meta-analysis, we found that age, degree of differentiation, tumor stage, and MDT were prognostic factors of colorectal cancer. These results were similar with previous studies [29, 31].

A population-based study reported the postoperative mortality after resection for colorectal cancer that was related to advanced age and tumor stage [38], and another population-based study reported similar results [39]. In this meta-analysis, we explored whether MDT had effect on the postoperative mortality, and no difference was found between MDT and non-MDT groups. Therefore, MDT might not affect postoperative mortality; although, accurate clinical data and imaging were accessed [40].

Although surgery played an important role in colorectal cancer, the requirement for cooperation of experts from various disciplines was increasing [41]. At present, most doctors in the world recognized the positive effect of MDT and accepted MDT as a main treatment for cancer [42]. MDT has demonstrated advantages not only in the field of digestive tract tumors but also in various fields of oncology. Many experts were involved in MDT and could easily deal with conflicting viewpoints, and this meant all experts could participate in a personalized tumor treatment based on clinical data and imaging. In this meta-analysis, after pooling the HRs, the MDT group was associated with better OS compared with the non-MDT group. In subgroup analysis of stage IV colorectal cancer, the MDT group was associated with better OS as well. There were many factors accounting for the current results. MDT could reduce the number of imperfect decisions made by individual physicians [12]. Moreover, patients could benefit from MDT including highly accurate staging, good treatment cohesion, high quality of life, and long-tem survival [31–33].

MDT could bring survival benefits, and the model of MDT had been in progress and explored [28]. The MDT meeting was challenging for involved experts, and self-education was important for surgeons, radiologists, pathologists, and oncologists participating in the meeting [28, 29]. However, van der Vlies et al. raised up a concern about negative outcome of MDT on OS, which was related to implementation of a preoperative MDT on patient management [18]. But if no adequate investment in team training, MDT might not be able to bring benefits to patients and health-care professionals [19].

Some limitations existed in this meta-analysis. Firstly, RCTs were not included in this meta-analysis, and only retrospective studies and cohort studies that subjected to inherent bias were included. Secondly, most of the studies included were from Asia, and the results might be applied to Asian patients. Thirdly, significant heterogeneity existed among the studies, and it seemed unlikely that any hospital system did multidisciplinary team therapy and then abandoned it, which meant that patients treated before the adoption of such teamwork and then incorporated into the study that might have received care in an earlier era when treatment options were less advanced. Alternatively, patients with truly poor prognosis or multiple comorbidities might not have been referred to the multidisciplinary team, which would have resulted in a selection bias. Although the effects of heterogeneity were reduced using the random-effect model method, this effect could not be completely eliminated. Furthermore, the included studies did not explicate the composition of MDT in detail, and the participation criteria of MDT remained unclear. Therefore, multicenter, multiregional, prospective, and high quality RCTs should be carried out in the future.

In conclusion, MDT could improve overall survival of colorectal cancer patients and had no influence on postoperative mortality.

## Figures and Tables

**Table 1:** Characteristics of the studies included in the meta-analysis.

Author	Year published	Country	Study design	Study date	Sample size		Gender (male/female)		Tumor staging		NOS
					MDT	Non-MDT	MDT	Non-MDT	MDT	Non-MDT	
van der Vlies	2020	The Netherlands	Cohort	2015-2018	127	306	65/62	191/115	I/II/III/IV	I/II/III/IV	8
Li	2020	China	Retrospective	2014-2018	46	83	32/14	54/29	IV	IV	7
Lin	2018	China	Retrospective	2006-2009	326	325	155/171	181/144	I/II/III/IV	I/II/III/IV	8
Hsu	2016	China	Cohort	2005-2008	3515	22251	1978/1537	12609/9642	I/II/III/IV	I/II/III/IV	8
Ye	2012	China	Cohort	1999-2006	298	297	166/132	180/117	I/II/III/IV	I/II/III/IV	8
Chien	2018	China	Retrospective	2007-2017	75	86	42/33	43/43	IV	IV	7
Lan	2016	China	Retrospective	2001-2010	439	636	261/178	429/207	IV	IV	8
Rogers	2017	Australia	Retrospective	2009-2012	363	257	227/136	140/117	I/II/III/IV	I/II/III/IV	7
Wille	2013	Denmark	Cohort	2001-2006	344	467	193/151	245/222	I/II/III/IV	I/II/III/IV	8
Du	2011	China	Retrospective	2001-2009	101	162	57/44	88/74	I/II/III/IV	I/II/III/IV	7
Macdermid	2009	The United Kingdom	Cohort	1997-2005	134	176	62/72	96/80	II/III/IV	II/III/IV	7

Abbreviations: MDT: multidisciplinary team; NOS: Newcastle-Ottawa Scale.

**Table 2:** Factors analysis of hazard ratios for overall survival.

Factors	No. of studies	Effects model	HR (95% CI)	*p*	Heterogeneity	
					*I* ^2^ (%)	*p*
Sex	5	Random	1.24 (0.93-1.66)	0.14	64	0.02
Age	5	Random	1.38 (1.09-1.74)	0.006	53	0.07
Neoadjuvant chemotherapy	2	Fixed	0.70 (0.45-1.10)	0.12	0	0.59
Differentiation	3	Fixed	1.35 (1.06-1.72)	0.01	47	0.15
Tumor stage	2	Random	1.77 (1.19-2.65)	0.005	87	0.006

Notes: HR: hazard ratio; 95% CI: 95% confidence interval.

## Data Availability

All data included in this study are available upon request by contact with the corresponding author.
